# Insights into the system of care of the elderly with mental disorders from the perspective of informal caregivers in Lithuania

**DOI:** 10.1186/s13033-019-0311-x

**Published:** 2019-08-13

**Authors:** Ausrine Kontrimiene, Jolanta Sauseriene, Ida Liseckiene, Leonas Valius, Lina Jaruseviciene

**Affiliations:** Department of Family Medicine, Lithuanian University of Health Sciences (LUHS), Mickeviciaus 9, LT 44307 Kaunas, Lithuania

**Keywords:** Primary health care, Mental health services, Social services, Informal care, Patient care, Elderly, Mental disorder

## Abstract

**Background:**

Changes in the demographics and respective growth of life expectancy and social needs make informal caregiving crucial component of comprehensive health and social care network, which substantially contributes to the health and well-being of the elderly. The purpose of this paper is to understand the system of care of elderly patients with mental disorders from the perspective of informal caregivers in Lithuania.

**Methods:**

We conducted five semi-structured focus group discussions with 31 informal caregivers attending to elderly patients with mental disorders. The data were audiotaped and transcribed verbatim. A thematic analysis was subsequently performed.

**Results:**

Five thematic categories were established: (1) the current state of care-receivers: representation of the complexity of patients’ physical and mental condition. (2) The current state of caregivers: lack of formal caregivers’ integration as a team; inadequate formal involvement of informal caregivers. (3) Basic care needs: the reflection of the group needs relating directly to the patient, care organisation and the caretaker. (4) The (non-) Readiness of the existing system to respond to the needs for care: long-term care reliance on institutional services, lack of distinction between acute/immediate care and nursing, lack of integration between the medical sector and the social care sector. (5) Potential trends for further improvement of long-term care for the elderly with mental disorders.

**Conclusions:**

Strengthening of the care network for elderly patients with mental disorders should cover more than a personalised and comprehensive assessment of the needs of patients and their caregivers. Comprehensive approaches, such as formalization of informal caregivers’ role in the patient care management and planning, a more extensive range of available services and programs supported by diverse sources of funding, systemic developments and better integration of health and social care systems are essential for making the system of care more balanced.

## Introduction

Demographic shifts occurring in western societies create rising tensions on the socio-economic, healthcare and social care areas due to the ageing of the population [1]. Advancing age is a major risk factor for dementia or Alzheimer’s disease, and, considering the prediction that by 2050 one-third of Europe’s population will be over 65 years old, special focus has been put on elderly patients with mental disorders both in Europe and in other regions [2, 3]. Different countries are developing various models of collaborative care, including those focused on informal caregiving, in order to strengthen the delivery of primary care and home healthcare services for elderly patients with mental disorders [4]. About 80% of older adults given primary health care have multiple chronic conditions including mental health problems, therefore their care is not limited to ensuring good health indicators and becomes a complex of care areas that include social care and informal care [5, 6].

Changes in demographics, a longer life expectancy, growth of the needs of patients make informal caregiving a crucial component of comprehensive health and social care network by substantially contributing to the health and well-being of the elderly [7]. Elderly with dementia enhances the need for informal care: seniors with normal cognitive function require on average 4.6 h of informal nursing assistance per week, whereas people with mild dementia need an additional 8.5 h of informal care per week and those with moderate to severe dementia respectively 17.4 and 41.5 additional hours of informal care per week [8].

The formal network of the health and social care systems is complicated and depends on the specific different legal frameworks, policies and separate financing [9]. Navigation through this network is challenging to the patients, their caregivers, and to health care professionals [10]. Eventually, teamwork and collaboration between the health and social care sectors become challenging: investigation of the cross-sector service delivery in health and social care highlights the necessity for delineating existing facilitators and barriers in cross-sector collaboration and use them to shape a common vision of care across different sectors [11].

Fragmentation of health and social care is also an issue in Lithuania where there is a lack of collaboration between family physicians and social workers [10]. It is especially problematic in the context of financial strain—spending’s on social protection per person in Lithuania reach nearly half of the EU average [12]. Research has shown that legislative attempts to foster collaboration between these sectors had a low impact on service provision and left organisational frameworks unchanged [13, 14]. Moreover, research has also proved that in the care for mentally ill patients in PHC the level of collaboration within the healthcare sector in Lithuania was very low [10].

Ineffective collaboration and inadequate coordination of care activities could negatively affect the well-being and safety of mentally ill elderly patients and impose an additional burden on their informal caregivers [15, 16]. However, a survey of the available research findings indicates that there have been no earlier studies of the experience of informal caregivers of mentally ill patients on this issue, nor has there been any attempt to understand the system of care for the mentally ill elderly in Lithuania. Therefore, this study aims to explore the experience of informal caregivers attending to the elderly with mental disorders and achieve a better understanding of the current system of care available to them.

## Methods

The research described in this paper represents the qualitative component of the project titled “*Integrated Health Care for Senior’s Mental Health: Developing an Intersectoral Cooperative Care Model”.* The 3-year project (2017–2020), financed by the Lithuanian Research Council (S-MIP-17-121), aims to assess the potential for collaborative work of primary health care (PHC), primary mental health care and social care services in Lithuania in order to better respond to the health and social care needs of families caring for elderly patients with mental disorders. The scope of this paper is to understand the system of care in Lithuania from the perspective of informal caregivers attending to elderly patients with mental disorders.

Regional Committee on Biomedical Research Ethics of Kaunas approved this study on 2018-04-23 (No:BE-2-47).

### The context of the health care system in Lithuania

Lithuanian primary health care is provided by public and private institutions. There are several types of care providers who are formally recognized as PHC professionals: general practitioners, community nurses, psychiatrists, mental care nurses, and social workers. There are two types of PHC teams working independently: general practitioners and community nurses working together in PHC centres as PHC teams; social workers, psychiatrists and mental care nurses working in primary mental health care centres as primary mental health care teams.

Medical care in Lithuania is free of charge to all senior patients, while additional fee is always present in the social care sector. There are two established nursing care levels of special needs for the elderly patients, which include financial (cash) benefits for the care receiver, full coverage of medication costs, special home equipment and accommodation. The aid is more focused on the care receiver, meanwhile the caregiver does not get any specific support, such as respite care or direct cash benefits [4].

### Study design

The scope of this paper is confined to data obtained from the study of focus groups of informal caregivers in Kaunas, which is Lithuania’s most central and highly urbanised region and constitutes 15% of Lithuania’s total population. General practitioners and psychiatrists from two large public primary health care institutions in Kaunas were informed about the aim of the project and were asked to notify the informal caregivers of mentally ill elderly patients about the study. Both institutions cover about 60% of Kaunas population (one institution has five different large divisions situated throughout Kaunas city). The informal caregivers who agreed to be contacted were put on a list (98 individuals) and later received a phone call from the researcher in order to explain the purpose and the course of the study and were invited to take part in the focus group discussions. A large proportion of the contacted caregivers (46) refused to take part in the study indicating that they could not leave their care receivers for more than an hour and had no backup caregiver; meanwhile, other caregivers have indicated different reasons (e.g. language barrier).

### Participants

A total of 31 informal caregivers participated in this study. Table 1 represents the participant’s sociodemographic details. The age varied from 34 to 74 years old (interquartile range (IQR) from 48 to 58 years of age). The majority of participants were female (n = 28) and three caregivers were male. More than half of the caregivers were children of care recipients (20 daughters and two sons). Time of caring ranged from 1 to 26 years (IQR from 2.5 to 8 years of care). The participants of the study provided written informed consents before the discussions and were guaranteed confidentiality; the usage of data to be collected during the study was also explained.

**Table 1:** Sociodemographic data of the study participants

The age of caregiver (years)	Sex of caregiver (M—male; F—female)	Relation with the patient	Years of care
48	F	Daughter	3
52	F	Daughter	2.5
55	F	Daughter	8
55	F	Daughter-in-law	3
49	F	Daughter	3
72	M	Son-in-law	4
61	F	Daughter	26
54	M	Son	1
42	F	Niece	11
53	F	Niece	1.5
58	F	Daughter	3
58	F	Daughter	5
59	F	Daughter	1.5
47	F	Daughter	2.5
40	F	Daughter	10
58	F	Daughter	8
34	F	Granddaughter	10
68	F	Daughter	13
74	F	Not a relative	9
41	F	Daughter	2
56	F	Niece	2
47	F	Daughter	5
52	F	Daughter	4
57	F	Daughter	5
62	F	Daughter	3
38	F	Granddaughter	2
52	F	Daughter	2.5
53	M	Son	4
58	F	Daughter	9
58	F	Daughter	2
58	F	Niece	7

### Data collection

Two facilitators trained in qualitative research methodology moderated each discussion. Five focus group discussions with 5 to 7 participants were conducted, and each lasted from 50 to 75 min (65 min on average). They were audio-taped with the participants’ permission. Each participant signed an informed consent form and confidentiality was assured as well.

A semi-structured topic guide was created for the focus group discussions (Table 2), which included open-ended questions about informal caregivers’ perceptions of the persons involved in outpatient care, the strengths and weaknesses of the present caregiving, the basic needs for proper care situation and their personal experiences as caregivers. The moderator encouraged experiential narratives. Each focus group discussion contained the same structure and core areas, but the course of each discussion varied depending on the participants’ insights. The content of the fourth focus group discussion was similar to the first three ones and the fifth focus group did not provide new content, thus we decided that the saturation of the theme has been reached and stopped the gathering of the data.

**Table 2:** Focus group discussions semi-structured topic guide

1. What type of specialists/individuals could you name as directly involved in the care/supervision or coordination of the one you care for? Please, describe what these people do. How are they involved in the care process? What is your experience regarding communication/collaboration with these specialists/individuals*?*
2. How would you evaluate the present care of the person you deliver care to—could you name the positive and the negative aspects? Could you tell us about the aspects that you are satisfied with? Could you tell us about the aspects of care you are struggling with?
3. In your opinion, what are the basic needs of your care receiver that should be met in order to ensure proper, more effective and better care overall? According to your experience, is it possible to satisfy those needs by using external resources? To what extent do you use personal resources (which ones) to meet those needs?
4. What is your experience as a caregiver? *How do you feel taking care of your family member?* *Who supports and strengthens you in this situation?* *What kind of help would you need as a caregiver?* *What are your personal expectations and needs?*
5. Maybe some other aspects have not been reviewed and you think they are important to be mentioned?

### Analysis

Each focus group discussion was transcribed word by word after all five focus group discussions had been completed. We used the thematic data analysis methodology. Two independent researchers analysed the data by using the inductive approach and reading the transcripts line by line. They created codes for selected terms, which were as close as possible to the words used by the participants themselves. Eventually, the codes were systematically compared, and the majority of them were similar. Discrepancies were discussed among the researchers until the consensus was reached. A combination of similar and related codes formed thematic categories, and the final themes were based on them. All of the themes and categories were reviewed several times by the researchers. Discussion quotations were used to illustrate each category. The label at the end of each illustration indicates the source of the quote (e.g., “G2” denotes the second focus group), bracketed ellipsis […] has the meaning of an omitted sentence. An unbracketed ellipsis indicates a reflective pause. Brackets were also used to indicate researchers comments for the clarification of the participant’s speech.

This manuscript analyses the informal caregivers’ experiences related to the system of care of elderly patients with mental disorders.

## Results

The data analysis enabled the researchers to identify five thematic categories: the current state of care-receivers; the current state of caregivers; basic care needs; the (non-) readiness of the existing system to respond to the needs for care; trends for further improvement.

### The current state of care-receivers

The focus group participants highlighted the complexity of conditions of patients to whom care is delivered: in most cases these are multiple conditions, both physical and mental. The number of supervised patients is another important issue—“*It is impossible to have three persons for nursing all by yourself.”* G2

According to the experience of informal caregivers, the daycare of patients depends on their confusion level, with the main challenges being related to their personal care, such as incontinence or refusal to drink and/or eat (or the opposite—excessive/disinhibited eating), to take medication or to go outdoors. The range of everyday activities of patients is limited, often centering around a certain place and people. The study participants shared the feeling of constant anxiety due to patients’ potency to harm themselves or others: physical violence, the danger of fire, the risk of poisoning:

*“For example, I even have such fears that she would eat something that she does not need. […] I found a cup which had soy sauce mixed with Fairy [dish washing detergent] in it. […] She is imagining some of her invisible guests enjoying the treat […] She slices some soap with a knife for them. Well, maybe she sees food or something in everything around, and at this point, it is scary for me.” G5*

### The current state of caregivers

According to focus groups participants, the network of outpatient caregiving relies mainly on family members and relatives.

*“I do not need anyone to help me, at least for me, thanks to my husband. We help each other. I nursed his mother for nine years, and now he helps me to do the same.” G5*

The informal caregivers have full integration of their personal lives and the task of administering the care.

*“Once in my life I said this sentence and it seems that it turned against me. “I will not live their lives, I have my own life”. However… now I see that I am living their lives.” G2*

Formal caregivers seem to be involved in care mostly in response to the expressed demand by informal caregivers. The study participants pointed out inadequate integration with the formal caregivers’ team and lack in communication, which is usually happening only on the informal caregiver’s demand—“*it would be perfect if they would just get in touch” G1.*

According to the study participants, the family physician and the community nurse are the first-contact in case of a new problem; meanwhile, the psychiatrist and the mental health nurse are usually providing prescriptions for medication. The study participants rarely mentioned social workers’ involvement in the delivery of care—most of the time the involvement of social workers was limited to the bathing of the patient and procurement of medicines.

*“There is a social worker who helps me bathe her, but she would not be useful for anything else, although the patient likes it when someone takes care of her.” G1*

### Basic care needs

During the focus group discussions, the participants shared their perceptions about everyday care needs and we grouped their insights into three clusters: needs directly related to the patient, to the care organisation and to the caregiver.

*The needs related to the patient* reflected informal caregiver’s attempts to ensure physical and psychosocial wellbeing of the patient. The study participants emphasized the need for a regular check-up including blood tests, periodic reassessment of treatment with the emphasis on the importance of physiotherapy and kinesiotherapy. In terms of the patient‘s psychosocial wellbeing, the informants pointed out the necessity to maintain activities that could ensure the pleasure component in patients‘ lives, naming specifically occupational therapy and meeting the patients‘ communication need.

*“I want only one thing, that someone would be there… to communicate. That she would not feel alone during the day.” G2*

*The needs related to the organisation of care* included the expectations to receive assistance when providing everyday care for the patients, ranging from ergotherapeutic home adaptation, assistance with transportation, provision of home-based nursing services to a larger supply of nursing care supplies (e.g. diapers).

*“In fact, there was a lack of home*-*based environmental adaptation. The grandmother would burn up food constantly while she still tried to cook food. Well, there were several times when there was a danger of a fire. So, dad constructed a timer that switches off electricity at a certain moment. […] The house was not adapted. The carpets were taken away by my parents because they [grandparents] started to stumble upon those carpets and fall.” G4*

*The caregivers’ need* included both expectations of psychological support as well as the available training to the caregiver. The focus group participants expressed the need for a comprehensive (systematic) assessment of the situation, including the conditions of both the patient and the caregiver, in order to map the strengths and weaknesses in each particular case. Then, they would welcome information about existing resources that would be indicated in each specific case.

*“In fact, there should be a person who would come to assess the situation from the outside, who knows about these issues. I think that such a person is needed. Because you do not even imagine what you need. How do you know what you need?” G2*

Moreover, they would prefer to have some formal training as caregivers instead of gaining practice on the job. The training would have to involve: acquisition of competency by evaluating existing resources and needs of care; the understanding of illness progression and related changes; communication and behaviour management of the patient; the basics in nursing and other.—“*you should know the basic things.” G2*

*“I would feel more at peace if I had a better understanding of, for example, how [the disease] progresses. How does this person react? Only after it [the disease] began, my doctor said, have you read that book…? I read it, I could hardly bring myself to read it, but I did read it.” G3*

*“When we were at the hospital […]they said to look at her tongue. Do you know this kind of thing? […]. The tongue shows that there is a lack of fluids.” G2*

Finally, focus group participants expressed their desire to be appreciated for the work they are doing—” *[…] our work is tough.” Both psychologically and physically… and financially.” 4G.* Acknowledgement and psychological support from their immediate family as well as from the formal caregivers seems to be vital in such situations—”*I am glad when someone calls, when someone shows concern.” G3.*

### The (non-) readiness of the existing system to respond to the needs for care

The experience of study participants revealed several characteristics of formal long-term care organisation: the care relied mainly on institutions with limited mobile services unable to provide adequate care at patient’s homes, lack of proactivity and anticipation of informal caregiver’s demands and lack of integration between the sectors of medical and social care.

In the participant’s points of view, nursing hospitals (long term care) and hospitals are the only providers of medical care outside the patient’s homes. Nursing hospitals serve mostly for respite care while hospitals respond to acute deterioration in health condition. However, it seems that the hospital care network lacks respect and empathy for elderly patients *“why are you pulling around that old man?” G3*. It is noteworthy that there is no agreement as to which hospital should take responsibility for acute care of elderly patients.

*“*-*We were driving around. I had to drive a lot with my father*- *from Kaunas Clinical Hospital to the Red Cross Hospital, to Kaunas Clinics until it was decided where he should be admitted.*

-*You are lucky that they admitted him. Mine was with embolisation and they did not admit him at first, only later… and he died. No one wanted to admit him. They told me that he had a hysteria attack. Well, he did have a strong leading character. So later it turned out that it was embolisation.” G4*

### Secondly, hospitals are not adequately prepared to provide health care for patients with mental disorders

*“Nobody [in hospitals] wants such things. If there is a psychiatric condition that is not completely in order… […], so no one wants them anywhere…” G1*

Moreover, hospitals face considerable difficulties when arranging a safe stay of a dependent patient without the escort of informal caregivers.

*“Here is another moment. He [the grandfather] was admitted and we still had to go there every day three times a day to look after him*- *the dad, me and my brother*- *we had to feed him, to watch him there… […] I think he was bedridden at that time. In that sense*- *he was taken to the hospital, and you are hoping that he will be taken care of. They did take care a little bit, but anyway we had to go there to feed him, to check if the diaper had been changed.” G4*

The participants of the study demonstrated poor understanding of the social care sector and it was mostly limited to the perceived system’s lack of capabilities and insufficient responsiveness: *“what can you expect there?” G4; “the system is complicated, I do not understand anything.” G5* or to the negative experience:

*“I went to social services for some help, after all, and they said that we do not know anything, we cannot help you with anything here… go to your family doctor.” G5*

It seems that such circumstances generate mistrust in the adequacy of the measures and procedures employed and evoke concern about a potential deterioration of the situation.

### Trends for further improvement

The following ideas for further development of care for the elderly with mental disorders emerged:

- Better integration of health and social care systems:
*“In fact, there could be, and I am just fantasizing, but there could be a system when you carry your papers to NDNT [Disability and working capacity assessment office]. […] Well, if you succeed, you get that [need for nursing care act]. So, if NDNT handled all the process… let us say social needs belong to social services, so the social unit team immediately comes into evaluate the situation and everything is clear, they communicate with medical services later. However, we do not have that.”G2*
- More clarity in the specific acute and long-term inpatient care services for the elderly:
*“That age, well I do understand that the age is advanced, but if that person was relatively healthy before… He had pneumonia but recovered, so everything was well and he was feeling better. He lived, I do not know, about two years after that. However, later we saw that he was already… and you could not take him anywhere, because nothing good would happen, so then he died with dignity at home. There are acute situations and chronic ones…” G4*
- The flexibility of the system so that a smooth transition between different long-term care services be ensured:
*“Let us say that there are those four months in the nursing hospital if you want to be admitted there. Let us say […] if a person might want procedures such as massage, exercise, some activity, so […] maybe if there were some departments where you could take this person for a week or so. […] I am telling you that this would be very good. That the person could be admitted to recover a little bit.” G1*
- Enhancement of the perceived social value of caregiving for the elderly (similar to the valorisation of motherhood), formalizing of caregiving by introducing a specific occupation, increase in standards of competence by potentially formalized training of informal caregivers:
*“You know, I would want that… you go there and you learn that as a human being. You get the explanation […] They would train you. […] maybe this could even be obligatory. Well, we are trying really hard, running around. Moreover, who knows how other people are doing the care. Maybe one should get such medical training as if for the driving license [formal obligatory medical training to get the drivers license].” G2*
- Development of formal caregiving resources for the elderly by generating commercial revenue from the patient’s real estate:
*“I visited a retirement home in Austria if this is the topic. There they give their property to the state and start living at those homes for seniors. They live a fantastic life there. With full care and with all four meals consisting of five dishes, like eating in a restaurant. Also, you can design your room as you want. […] Children come to visit. Because everyone works. […] it is nice to watch. […] But they also said that they left their apartments to the state and not to their children.” G4*

## Discussion

The results of the study provide an insight into the issues of the system of care for mentally ill elderly patients in Lithuania from the perspective of informal caregivers. As informal caregiving mostly relies on family members, informal caregivers expect to get treatment as members of the care team through access to formal training and appreciation as professionals.

The informal caregivers interviewed in this study expressed their interest in having an expanded and better-integrated system of care with a more personalized and comprehensive assessment of the needs of both the patient and the caretaker, with a bigger variety of available services and programs supported by diverse sources of funding. The expectations of the focus group participants regarding a better-coordinated approach of health and social care professionals suggest an urgent need for better integration of these sectors; their experience in hospital care brings insight into the potential need for geriatric specialisation of hospital services. The findings of our study are in line with those other evidence-based studies about the complexity of health and social care systems. Efforts have to be taken to integrate these sectors more closely and meet challenges predetermined by different professional identities, cultures and philosophy of care [11].

The results of our study call for better coordination of social and health care sectors and higher “readability” and “user-friendliness” of the social care system and comply with other similar research findings [17]. Various models for improving care and addressing the needs of patients and their caregivers could be applied, such as the introduction of system navigators that would help to fill in the gap between health and social care needs in primary care [18, 19] or the involvement of social workers into primary care teams [11, 20]. In the literature there seems to be widespread support of the benefits of delivery of social work services provision in primary care for adults with complex social and health care needs as it demonstrates to have improved their subjective health, reduced barriers to treatment and helped maintain better health, self-management and functioning [21].

Recent changes in the Lithuanian legal framework have included social workers into the primary care team [22], which could favourably inspire the development of a comprehensive response to the needs of elderly patients with mental disorders. However, further efforts are to be taken to achieve smoother collaboration between the health and social sectors. The Canadian experience in intersectoral integration highlights the importance of strengthening communication among the various care providers, along with clarifying the role of social work in interdisciplinary care, furthermore, emphasises the need for adequate training and competencies, as well as having clear organisational structures [23].

The distinction between roles related to care delivery for elderly patients in general and elderly patients with mental disorders should become a priority target for the health sector. Experience of focus group participants reveals potential confusion and mistrust in the health care sector when acute cases of elderly patients with mental disorders are encountered. However, this might be attributed to the fact that geriatric care is in its initial stage of development in Lithuania. The development of the infrastructure of care infrastructure for the elderly is a priority in Europe and worldwide: the World Health Organisation released its mid-term progress report on “*Global strategy and action plan on ageing and health”* in 2017, which assessed the progress in the implementation of this strategy with ten progress indicators (e.g., focal points on ageing and health; plan on ageing and health; legislation and enforcement against age-based discrimination and other) [3]. Certain activities to the implementation of the strategy have been described in the report: specific guidelines for primary care practitioners on integrated care for the elderly, online training programs for caregivers, support for countries in delivering integrated care for the elderly and other preparations toward a new decade of healthy ageing [3]. Organisation for Economic Co-operation and Development (OECD) is encouraging to put the creation of national dementia care plans in the forefront for all the member countries, and emphasize that these plans need to be supported by systems of with sufficient funding and progress measurement [2].

Our data confirm the findings of other studies in that informal caregivers are an essential part of the ecosystem of care for elderly patients with mental disorders [17]. A systematic review of qualitative studies of caregivers needs of patients with dementia identified two essential areas: those related to the management of care of the elderly and those related to the personal needs of caregivers [24]. A significant potential for the improvement of care rests with further formal inclusion of informal caregivers in care teams. This would allow to better understand the needs of the patients as well as their caregivers, to more efficiently allocate resources and to prevent the reliance on institutional long-term care: proper caregiving on behalf of the family can delay placement in a nursing home for up to 1.5 years in moderate dementia. [25]. A comprehensive review of cross-sector service provision in health and social care emphasised the importance of involvement of patients and their families along with front-line healthcare professionals and policymakers in the development of a common vision of care across different sectors [11]. A large amount of research highlights several areas where caregivers can be supported in their work: information, short-term physical care, comprehensive counselling, home adaptation and equipment, support groups, psychoeducation, social support and flexible working conditions [24–26]. Interventions into care delivered by for informal caregivers show promising results in terms of stress and anxiety management caused by caregiver’s subjective burden [27–29].

Despite ongoing discussions in the scientific literature about the economic and financial variables involved in the health and social care provision of the elderly, scarce data is available to support specific conclusions about cost-effectiveness, though results are promising for cost containment, disease prevention, and health of population [9, 29–31]. A systematic review of respite for caregivers demonstrated that daycare is as costly as the usual care, but caregivers were satisfied and reported a positive effect on their mental or physical health [30]. A novel approach has been suggested where the focus would be centred on the added value of care rather than cost saving [29], however, a more profound and accurate scientific analysis of cost-effectiveness, cost–benefit or cost-utility is needed [31].

### Limitations

This study has several limitations. The informal caregivers were invited to take part in this study via their general practitioners or psychiatrists. There is a probability that if the study had included participants from the records of the social welfare sector, their exposure and input to the social care system would have been greater as compared to our focus group participants. However, in our study we aimed to elicit the experience of informal caregivers in the system of health care rather than to assess the users’ general awareness and accessibility of health care and social care systems.

There is another potential limitation of this study related to the participants. The final sample of the study participants was rather small, even though we contacted 94 eligible participants, only 31 of them participated in the study. Furthermore, the caregiver participation was inconsistent across the study: at each step, some informal caregivers were unwilling or unable to take part in the focus group discussions. As the majority of those who did not participate and ascribed their absence to inability to find coverage or replacement for their caregiving, it is highly probable that the study missed the reported experience that would have been more negative and concerning. Further qualitative studies, including semi structured or in-depth interviews, might be needed to elicit this subgroup’s experience.

## Conclusions

Strengthening of the care network for elderly patients with mental disorders should be focused on a more personalised and comprehensive assessment of the needs of patients and their caregivers. The social and health care sectors should consider formal involvement of informal caregivers in the management and planning of the patient care provision. Furthermore, they should consider not only the needs of the patient but the needs of the informal caregiver as well. Comprehensive approaches, such as a broader range of available services and programs supported by diverse sources of funding could facilitate the care provision for informal caregivers. A systematic development and better integration of the health and social care systems are essential in order to make a more balanced intersectoral collaboration between health and social care sectors.

## Data Availability

The datasets analysed during the current study are available from the corresponding author on reasonable request.

## Abbreviations

PHC: primary health care
OECD: Organisation for Economic Co-operation and Development
IQR: interquartile range

## References

[1] Alzheimer’s Disease International. The World Alzheimer Report 2018: the state of the art of dementia research: new frontiers. London: Alzheimer’s Disease International (ADI); 2018. https://www.alz.co.uk/research/WorldAlzheimerReport2018.pdf?2. Accessed 20 Mar 2019.

[2] Organisation for Economic Co-operation and Development (OECD). Renewing priority for dementia: where do we stand? Policy brief. OECD; 2018. http://www.oecd.org/health/health-systems/Renewing-priority-for-dementia-Where-do-we-stand-2018.pdf. Accessed 20 Mar 2019.

[3] World Health Organization. Global strategy and action plan on ageing and health. Geneva: World Health Organization; 2017. Licence: CC BY-NC-SA 3.0 IGO. https://www.who.int/ageing/WHO-GSAP-2017.pdf?ua=1. Accessed 20 Mar 2019.

[4] Zigante V. Informal care in Europe—exploring formalisation, availability and quality. European Commission; 2018. https://ec.europa.eu/social/main.jsp?catId=738&langId=en&pubId=8106&type=2&furtherPubs=no. Accessed 20 Mar 2019.

[5] O’Brien R, Wyke S, Guthrie B (2011). An’endless struggle’: a qualitative study of general practitioners’ and practice nurses’ experiences of managing multimorbidity in socio-economically deprived areas of Scotland. Chronic Illn.

[6] Palmer K, Marengoni A, Forjaz MJ, Jureviciene E, Laatikainen T, Mammarella F (2018). Multimorbidity care model: recommendations from the consensus meeting of the Joint Action on Chronic Diseases and Promoting Healthy Ageing across the Life Cycle (JA-CHRODIS). Health Policy.

[7] Bouget D, Spasova S, Vanhercke B. Work-life balance measures for persons of working age with dependent relatives in Europe. A study of national policies. European Social Policy Network (ESPN). European Commission; 2016. https://publications.europa.eu/en/publication-detail/-/publication/ef6ed9fd-9a96-11e6-9bca-01aa75ed71a1/language-en. Accessed 20 Mar 2019.

[8] Langa KM, Chernew ME, Kabeto MU (2001). National estimates of the quantity and cost of informal caregiving for the elderly with dementia. J Gen Intern Med.

[9] Rizzo VM, Rowe JM (2016). Cost-effectiveness of social work services in aging: an updated systematic review. Res Soc Work Pract.

[10] Jaruseviciene L, Sauliune S, Jarusevicius G, Lazarus JV (2014). Preparedness of Lithuanian general practitioners to provide mental healthcare services: a cross-sectional survey. Int J Ment Health Syst.

[11] Winters S, Magalhaes L, Anne Kinsella E, Kothari A (2016). Cross-sector service provision in health and social care: an umbrella review. Int J Integr Care.

[12] Eurostat [Internet]. European Commission; 2019. https://ec.europa.eu/eurostat/data/database. Social protection expenditure; 2019. https://ec.europa.eu/eurostat/data/database. Accessed 20 Mar 2019.

[13] Jaruseviciene L, Liseckiene I, Valius L, Kontrimiene A, Jarusevicius G, Lapão LV (2013). Teamwork in primary care: perspectives of general practitioners and community nurses in Lithuania. BMC Fam Pract.

[14] Groenewegen PP, Dourgnon P, Greß S, Jurgutis A, Willems S (2013). Strengthening weak primary care systems: steps towards stronger primary care in selected Western and Eastern European countries. Health Policy.

[15] Oster C, Henderson J, Lawn S (2016). Fragmentation in Australian Commonwealth and South Australian State policy on mental health and older people: a governmentality analysis. Health (London).

[16] Kirst M, Im J, Burns T (2017). What works in implementation of integrated care programs for older adults with complex needs? A realist review. Int J Qual Health Care.

[17] Courtin E, Jemiai N, Mossialos E (2014). Mapping support policies for informal caregivers across the European Union. Health Policy.

[18] Carter N, Valaitis RK, Lam A, Feather J, Nicholl J, Cleghorn L (2018). Navigation delivery models and roles of navigators in primary care: a scoping literature review. BMC Health Serv Res.

[19] Robinson-White S, Conroy B, Slavish KH, Rosenzweig M (2010). Patient navigation in breast cancer: a systematic review. Cancer Nurs.

[20] Fraser M, Lombardi B, Wu S, Zerden LS, Richman E, Fraher E (2018). Social work in integrated primary care: a systematic review. J Soc Soc Work Res.

[21] McGregor J, Mercer SW, Harris FM (2018). Health benefits of primary care social work for adults with complex health and social needs: a systematic review. Health Soc Care Community.

[22] Organisation of the primary ambulatory care services and procedure for payment of these services expenses. [Dėl pirminės ambulatorinės asmens sveikatos priežiūros paslaugų teikimo organizavimo ir šių paslaugų išlaidų apmokėjimo tvarkos aprašo tvirtinimo]. Decree of Minister of Health of Lithuania [Lietuvos respublikos sveikatos apsaugos ministro isakymas], 2019 January 24; No V-101, Vilnius. https://e-seimas.lrs.lt/portal/legalAct/lt/TAD/53d0bc6022e311e9b246d9cc49389932. Accessed 20 Mar 2019. (**in Lithuanian**).

[23] Ashcroft R, McMillan C, Ambrose-Miller W, McKee R, Brown JB (2018). The emerging role of social work in primary health care: a survey of social workers in Ontario family health teams. Health Soc Work.

[24] McCabe M, You E, Tatangelo G (2016). Hearing their voice: a systematic review of dementia family caregivers’ needs. Gerontologist.

[25] Mittelman MS, Haley W, Roth D (2006). Improving caregiver well-being delays nursing home placement of patients with Alzheimer’s disease. Neurology.

[26] Zwingmann I, Michalowsky B, Esser A (2019). Identifying unmet needs of family dementia caregivers: results of the baseline assessment of a cluster-randomized controlled intervention trial. J Alzheimers Dis.

[27] Sherifali D, Ali MU, Ploeg J (2018). Impact of internet-based interventions on caregiver mental health: systematic review and meta-analysis. J Med Internet Res.

[28] Weinbrecht A, Rieckmann N, Renneberg B (2016). Acceptance and efficacy of interventions for family caregivers of elderly persons with a mental disorder: a meta-analysis. Int Psychogeriatr.

[29] Steketee G, Ross AM, Wachman MK (2017). Health outcomes and costs of social work services: a systematic review. Am J Public Health.

[30] Mason A, Weatherly H, Spilsbury K, Arksey H, Golder S, Adamson J (2007). A systematic review of the effectiveness and cost-effectiveness of different models of community-based respite care for frail older people and their carers. Health Technol Assess.

[31] Easton T, Milte R, Crotty M, Ratcliffe J (2017). Where’s the evidence? a systematic review of economic analyses of residential aged care infrastructure. BMC Health Serv Res.

